# Levels and Determinants of COVID-19 Vaccination at a Later Phase among Chinese Older People Aged 60 Years or Older: A Population-Based Survey

**DOI:** 10.3390/vaccines11061029

**Published:** 2023-05-26

**Authors:** Yanqiu Yu, Stefanie Hoi Ying Yen, Li Crystal Jiang, Wai-kit Ming, Mason M. C. Lau, Joseph T. F. Lau

**Affiliations:** 1 Department of Preventive Medicine and Health Education, School of Public Health, Fudan University, Shanghai 200032, China; 2 Health Communication Institute, Fudan University, Shanghai 200032, China; 3 Centre for Health Behaviours Research, Jockey Club School of Public Health and Primary Care, The Chinese University of Hong Kong, Hong Kong SAR, China; 4 Department of Media and Communication, City University of Hong Kong, Hong Kong SAR, China; 5 Department of Infectious Disease and Public Health, Jockey Club College of Veterinary Medicine and Life Sciences, City University of Hong Kong, Hong Kong SAR, China; 6 Zhejiang Provincial Clinical Research Center for Mental Disorders, The Affiliated Wenzhou Kangning Hospital, Wenzhou Medical University, Wenzhou 325000, China; 7 School of Mental Health, Wenzhou Medical University, Wenzhou 325000, China; 8 School of Public Health, Zhejiang University, Hangzhou 310058, China

**Keywords:** COVID-19 vaccination, elderly people, health perceptions, interpersonal influences, health information, early vaccination

## Abstract

The early attainment of high COVID-19 vaccination rates can minimize avoidable hospitalizations/deaths. The fifth wave COVID-19 outbreak in Hong Kong caused >9000 deaths, and most of them were unvaccinated older people. This study hence investigated determinants of taking the first dose vaccination at a later phase (Phase 3: during the fifth wave outbreak, i.e., February–July 2022) versus two earlier phases (Phase 1: first six months since vaccine rollout, i.e., February–July 2021; Phase 2: six months prior to the outbreak, i.e., August 2021–January 2022) via a random telephone survey among 386 ever-vaccinated Hong Kong older people aged ≥60 (June/July 2022). A total of 27.7%, 51.1%, and 21.3% took the first dose at Phase 1, Phase 2, and Phase 3, respectively. Unfavorable perceptions related to COVID-19/vaccination, exposure to conflicting/counter-information about the suitability of older people’s vaccination from various sources, unsupportive family influences prior to the outbreak, and depressive symptoms were significantly associated with taking the first dose at Phase 3 instead of Phase 1 and Phase 2. To speed up COVID-19 vaccination and avoid unnecessary deaths, the government and health professionals should rectify misinformation, provide clear/consistent information for older people and their family members, and focus on those having depressive symptoms at an earlier stage of the pandemic.

## 1. Introduction

The COVID-19 pandemic has prevailed since March 2020. Vaccination is considered to be the most effective means of controlling the pandemic [1,2]. Evidence has clearly demonstrated that COVID-19 vaccination can effectively reduce hospitalizations and deaths [3,4]. Notably, the vaccination rate has exceeded 80% in a number of countries such as South Korea, Japan, Spain, Chile, and China [5]. In contrast, vaccination intention studies conducted in some countries prior to their rollout periods have commonly recorded high vaccine hesitancy and low prevalence of intention of vaccination [6,7,8,9,10,11,12]. The apparent ‘paradox’ between the previously high pre-rollout vaccine hesitancy versus high eventual vaccination rates reported in the literature might have been caused by changes in epidemiology, policy [13], and perceptions [14] over the pandemic period. Another explanation is that many people demonstrating hesitancy at early rollout phases were in fact observing and assessing the benefits versus safety of COVID-19 vaccination [15]; they were hence laggards according to the Diffusion of Innovation Theory [16] instead of being refusers of COVID-19 vaccination. For instance, a study conducted in Hong Kong in September 2020 demonstrated that 68.2% of the adult general population were holding a wait-and-see attitude [15]. Notably, vaccine hesitancy and refusal against vaccination are not equivalent [17].

As of 21 August 2022, there are over 6.4 million deaths related to COVID-19 globally [18]. Strong evidence has consistently demonstrated that the risk of death among unvaccinated versus vaccinated COVID-19 patients was multi-fold (e.g., 11 times) [19]. Globally, unvaccinated deaths are numerous. Therefore, the high vaccination rate of a country needs to be attained as early as possible. The timing of a completed vaccination at the population level is an under-emphasized element determining the devastation of the pandemic, as many deaths could have been saved if the ultimately high vaccination rate had been achieved within a shorter time frame. In future pandemics of respiratory infections, the public health concern should not only focus on high ultimate vaccination rates, but also on when such high rates could be achieved. Attention about individuals’ timing of vaccination, or specifically, why people did not vaccinate in the early phases of the COVID-19 vaccine rollout, has significant yet unexplored implications. Such research is warranted.

Furthermore, COVID-19 vaccination campaigns need to target specific age groups, as older age is an important factor of both COVID-19 vaccination [20] and COVID-19 deaths [21,22]. Deaths can in turn be reduced by vaccination [3,4]. Epidemiologically, older COVID-19 patients (e.g., those older than 60) were more likely than their younger counterparts to die from COVID-19 [23]. Vaccination among older people is hence a serious global health concern. The relationship between older age and COVID-19 vaccination, however, varies across populations. In countries such as the U.S., the U.K., Canada, and Japan, the vaccination rates in older age groups were higher than those of the younger groups [24,25,26,27]. The reverse was, however, true in some countries such as Mainland China, Romania, and Latvia [28,29]. Specifically, as of 31 December 2021, the prevalence of vaccination by age was 70.0%, 79.7%, 62.9%, 45.6%, and 20.6% for the age groups of <50, 50–59, 60–69, 70–79, and ≥80, respectively, in the community-dwelling adults in Hong Kong [30,31]. Even in some countries with relatively high vaccination rates, the absolute number of unvaccinated older people remains substantial.

This study investigated the distribution of the timing of COVID-19 vaccination in three 6-month phases since the rollout of free vaccination among vaccinated older people in Hong Kong. Phase 1 was the first six months since the rollout (February 2021 to July 2021); Phase 2 refers to the second 6-month period, which was about the 6-month period prior to the fifth wave outbreak (August 2021 to January 2022); Phase 3 refers to the 6-month period of the fifth wave outbreak period (February 2022 to July 2022). In Phase 1 and 2, Hong Kong was, in general, able to maintain zero new COVID-19 cases per day entirely for four months (from October 2021 to January 2022) [32]. Yet, in Phase 3, there was a sudden surge to over 50,000 new cases per day in early March 2022, and a total of 1.2 million new COVID-19 cases were reported from February to April 2022 [32]. Outbreaks of severe emerging infectious diseases may motivate people to take up vaccination [33,34]. According to the Health Belief Model (HBM), such outbreaks are likely to increase people’s perceived severity and perceived susceptibility, which are determinants of health behaviors [35] and were positively associated with COVID-19 vaccination behavior and intention [36]. Yet, the vaccination completion rate (at least two doses) for the ≥60 age group was only 48.3% as of 31 December 2021 and had increased sharply to 81.2% as of 22 August 2022 [30,31]. Hence, a substantial number of older people in Hong Kong had not taken up or completed COVID-19 vaccination prior to the fifth wave outbreak. It was tragic that despite the low number of COVID-19-related deaths reported in Hong Kong as of 1 February 2022, the fifth wave outbreak caused over nine thousand deaths [31], of which 6359 (about 70%) were unvaccinated older individuals aged ≥60 years [31]. Some of those deaths could have been avoided if a higher proportion of the older people in Hong Kong had taken up vaccination prior to the fifth wave outbreak. It is thus greatly warranted to understand factors affecting the timing of vaccination (vaccination in the early phases versus vaccination during the fifth wave outbreak) among those who have taken up COVID-19 vaccination.

This study hence investigated retrospectively how older people’s perceptions, exposure to unsupportive information about the suitability of vaccination, and unsupportive interpersonal influences at the earlier phase of the rollout (i.e., prior to the fifth outbreak) were associated with the timing of vaccination among vaccinated older people in Hong Kong. Regarding perceptions, first, it is likely that older people have negative attitudes toward COVID-19 vaccination, such as strong concerns over the safety of COVID-19 vaccination due to their age and chronic disease conditions [37]. There were wide-spread reports about vaccinated older peoples’ deaths soon after the rollout of COVID-19 vaccination in Hong Kong [38], which might have reduced older people’s motivation to take up COVID-19 vaccination. Second, since the pandemic was under relatively good control in Hong Kong prior to the fifth wave outbreak (with a low average of 10.7 new cases per day from February 2021 to January 2022 [39]), older people might perceive a low risk of infection, which might have reduced their motivation to take up vaccination in the early phase of the rollout, as perceived risk is known to be associated with COVID-19 vaccination [40,41]. News about post-vaccination deaths among older people were widely publicized at the early phases of vaccine rollout [38]. Such information might amplify the worries of severe side effects of the vaccines and defer vaccination [42].

The second group of factors was related to the exposure to unsupportive information about older people’s suitability for COVID-19 vaccination that was received at the earlier phase of the rollout period (prior to the fifth wave outbreak). Frequencies of receiving information about COVID-19 vaccination was positively associated with COVID-19 infection [42,43,44]. Yet, confusing and inconsistent information and misinformation about COVID-19 vaccination by local experts, the government, and the media/social media were common, especially during the earlier phase of the rollout period [38]. Counter information that older people should not take up vaccination might also have been given by some significant sources, such as health professionals [43] and social media [42,43]. Such information has also influenced vaccination behavior/intention [42,43]. Misinformation was a strong factor against COVID-19 vaccination [44].

Literature has demonstrated that older people’s health service utilization is often subjected to interpersonal influences, such as those arising from family members [45]. The same applies to COVID-19 vaccination [15]. Behavioral health theories have emphasized the importance of such influences. For instance, the Theory of Planned Behavior (TPB) postulates that the subjective norm (supportiveness of significant others) is an important determinant of health behaviors [46]. Positive associations between the subjective norm and COVID-19 vaccination have been widely reported in the literature [47,48]. Another interesting aspect of interpersonal influences is the involvement of family members in decision making regarding COVID-19 vaccination. Older people sometimes rely on their family members, especially younger ones, when making decisions regarding health-related problems such as treatments [49] and vaccinations [50], as they might believe that their family members are more educated and knowledgeable. It was hence hypothesized that unsupportive subjective norm and decision making involving family members during the earlier phase of the rollout would defer vaccination.

Mental distress in general and specific to COVID-19 was common during the entire pandemic period [51,52]. Research demonstrated that depression was negatively associated with COVID-19 vaccination [53], as depression may in general deprive motivation of health-related self-care [54] and seeking help [55]. Older people were vulnerable to depression during the pandemic period. Some research has reported a high prevalence of probable mild-to-moderate depression of 52.3% in the older population in Hong Kong [56]. The present study hence looked at the potential impact of depression on the timing of vaccination.

Given the background, this study investigated the prevalence of COVID-19 vaccination in three 6-month phases since the rollout of free vaccination (Phase 1, 2, and 3 as aforementioned) among vaccinated older people in Hong Kong. Four comprehensive groups of retrospective factors were investigated, comparing Phase 3 vaccination versus Phase 1 vaccination and Phase 3 vaccination versus Phase 2 vaccination. Such factors included (1) perceptions (negative attitude regarding COVID-19 vaccination and perceived risk of contracting COVID-19), (2) unsupportive information (exposure to news about post-vaccination deaths involving older people, conflicting information, and counter-information about the suitability of vaccination for older people), (3) interpersonal influences (unsupportive subjective norm and vaccination decision making involving family members), and (4) mental distress (depressive symptoms). It was hypothesized that the four aforementioned groups of factors would be associated with the likelihood of taking up COVID-19 vaccination at Phase 3 versus Phase 2 and Phase 3 versus Phase 1.

In addition, comparisons were made between those vaccinated prior to versus after the fifth wave outbreak, in terms of: (a) the perceived increase in worries about COVID-19 infection due to the fifth wave outbreak, (b) perceived increase in worries about COVID-19 related hospitalization or death due to the fifth wave outbreak, (c) perceived relationship between older COVID-19 patients’ death and the low vaccination rate among older people during the fifth wave outbreak, and (d) level of emotional distress when the fifth wave outbreak occurred.

## 2. Materials and Methods

### 2.1. Participants and Data Collection

The study population comprised Chinese people aged ≥60 years and having taken at least one dose of COVID-19 vaccination. “One dose” was chosen as the parameter, as most people taking one dose in Hong Kong had completed two doses [30] and as many people started taking the first dose during the fifth wave outbreak and hence would not have been scheduled for the second dose at the time of the survey. A random telephone survey was conducted from June 20 to 18 July 2022. Telephone numbers were randomly generated from updated fixed-line phone directories. Interviews were made from 5pm to 10pm (10 to 15 min) by experienced field workers to avoid over-sampling non-working individuals. The household member whose birthday was closest to the interview date and fulfilling the inclusion criteria were invited to join the study. Unanswered telephone calls were given at least three attempts before being classified as invalid. No incentives were given to the participants. Briefing and verbal informed consent were obtained from the participants. Ethics approval was obtained from the ethics committee of the corresponding author’s affiliated institution. 

A total of 957 valid telephone contacts were made. Four hundred (response rate: 400/957 = 52.2%) interviews were completed, 24 (6%) of which had not taken any dose of COVID-19 vaccination and were excluded from the data analysis. The effective sample size was 376.

### 2.2. Measurement

The questionnaire of this study was developed by a panel of three researchers who are experienced in public health, epidemiology, behavioral health, and health psychology. A thorough literature review was first conducted on factors of COVID-19 vaccination. Based on the results and the concepts of related variables, the panel constructed the items and rated the content validity by consensus. The items were then pilot tested on five adults aged 60 years or older (the target population) to test the face validity and readability. Based on the feedback, the panel finalized the items.

#### 2.2.1. Background Factors

Such information included age group, sex, education level, current marital status, employment status (full time/part time/retired/homemaker), whether living alone, chronic disease condition, and previous COVID-19 infection.

#### 2.2.2. Time Period in Taking the First Dose of COVID-19 Vaccination

Participants were classified into three groups according to the timing of their first dose of COVID-19 vaccination: (a) Phase 1 of the first six months since the rollout (i.e., February 2021 to July 2021), (b) Phase 2 of the second six months since the rollout, which was equivalent to the 6-month period prior to the fifth wave outbreak (August 2021 to January 2022), and (c) Phase 3 refers to the third six months, which was the fifth wave outbreak period (i.e., February 2022 to July 2022).

#### 2.2.3. Perceptions Prior to the Fifth Wave Outbreak

1) Negative attitude towards COVID-19 vaccination was assessed by a 2-item summative scale. The two items asked participants’ perceptions about (a) strong side effects of COVID-19 vaccination, and (b) low protectiveness of COVID-19 vaccination (1 = strongly disagree to 5 = strongly agree). The Cronbach’s alpha was 0.65 in this study.
2) Perceived low risk of COVID-19 infection was assessed by the item: “During the pre-fifth wave period, I believed that I am at low risk of contracting COVID-19” (1 = strongly disagree to 5 = strongly agree).

#### 2.2.4. Exposure to Unsupportive Information about Suitability of Older People’s Vaccination Prior to the Fifth Wave Outbreak

1) Conflicting information about suitability of older people’s vaccination was assessed by a 3-item summative scale, which asked about the level of agreement towards three statements regarding the existence of contradictory and confusing information regarding whether older people or those having chronic diseases were suitable to take up COVID-19 vaccination prior to that delivered by (i) local health experts, (ii) the local government, and (iii) mass/social media (1 = strongly disagree to 5 = strongly agree). A summative scale was formed (Cronbach’s alpha = 0.97).
2) Counter information that “older people and those having chronic disease should not take up COVID-19 vaccination” obtained from (i) media/social media, and (ii) health professionals (1 = strongly disagree to 5 = strongly agree).
3) Frequency of exposure to news reporting post-vaccination deaths of older people was assessed by one item (1 = none to 5 = always).

#### 2.2.5. Family Influences over COVID-19 Vaccination Prior to the Fifth Wave Outbreak

1) Family’s unsupportive attitude towards COVID-19 vaccination was assessed by the item: “Prior to the fifth wave outbreak, were your family members supportive or unsupportive of your taking up COVID-19 vaccination” (1 = strongly supportive to 5 = strongly unsupportive).
2) Involvement of family members in the vaccination decision-making process: Participants were asked who decided whether the participant would take up COVID-19 vaccination. Response categories included: mainly by the participant, jointly by the participant and family members, and mainly by family members.

#### 2.2.6. Depressive Symptoms

Depressive symptoms were assessed by using the 9-item Patient Health Questionnaire (PHQ-9), which is a commonly used screening tool assessing the severity of depression. The Chinese version of PHQ-9 has been validated and demonstrated satisfactory psychometric properties [57]. The items were rated with 4-point Likert scales on the frequencies of having potential symptoms (e.g., “feeling down, depressed, or hopeless”) in the past two weeks (0 = none at all to 3 = almost every day). Cut-off values of 5, 10, 15, and 20 were used to classify probable mild, moderate, moderately severe, and severe depression, respectively. The Cronbach’s alpha was 0.77 in this study.

#### 2.2.7. Perceptions and Emotional Distress Related to the Fifth Wave Outbreak

Participants were asked whether they agreed with the statements regarding perceptions and emotions related to the fifth wave outbreak, including (a) increase in worries about COVID-19 infection, (b) increase in worries about hospitalization or death due to COVID-19, (c) perceived strength of the relationship between older COVID-19 patients’ death during the fifth wave outbreak and low vaccination rate in the older population, and d) “How much emotional distress did you experience when the fifth wave outbreak occurred?” (1 = extremely low to 5 = extremely high).

### 2.3. Statistical Analysis

Two dependent variables about the timing (phases) of the first dose COVID-19 vaccination were used in this study, i.e., comparisons of Phase 3 versus Phase 2 (reference group) and Phase 3 versus Phase 1 (reference group). Univariable logistic regression analyses were conducted to test the individual associations between background factors/independent variables and these two dependent variables; crude odds ratio (ORc) and their corresponding 95% confidence interval (CI) were reported. Furthermore, with the adjustment of background factors (sex, age groups, education level, current marital status, employment status, whether living alone, and chronic disease status), multivariable logistic regression analyses (variable selection = Enter) were conducted to test the individual associations between independent variables and the two dependent variables; adjusted odds ratio (ORa) and their corresponding 95% CI were reported. The Chi-square test was performed to test the between-group differences in perceptions and emotions related to the fifth wave outbreak. SPSS Version 23.0 was used for statistical analyses. Statistical significance was defined as two-tailed *p*-value < 0.05.

## 3. Results

### 3.1. Descriptive Statistics

Close to or over half of the participants were female (65.2%), aged >70 year (44.5%), retired (46.5%), and having at least one of the listed types of chronic diseases (62.5%). About 10% had attained college or above education (8.5%) and were living alone (12.2%). About one quarter were not currently married (22.6%) and had experienced COVID-19 infection (25.8%). Regarding the timing of the first dose vaccination, 27.7%, 51.1%, and 21.3% took the first dose COVID-19 vaccination during Phase 1, Phase 2, and Phase 3, respectively. The descriptive statistics of the independent variables are presented in Table 1.

The mean (SD; range) scale/item scores of variables regarding situations prior to the fifth wave outbreak were 5.9 (1.6; 2–10) for negative attitude towards COVID-19 vaccination, 3.5 (1.0; 1–5) for perceived low risk of COVID-19 vaccination infection, 3.4 (0.7; 1–5) for frequency of exposure to news reporting older people’s post-vaccination deaths, 10.8 (2.9; 6–15) for conflicting information about older people’s suitability for COVID-19 vaccination, 3.3 (1.0; 1–5) for counter information about suitability of older people’s vaccination obtained from mass/social media, 2.3 (0.8; 1–5) for counter information about participant’s suitability of vaccination given by health professionals, and 3.6 (1.8; 0.8) for family’s unsupportive attitude towards COVID-19 vaccination. The mean (SD; range) score of depressive symptoms was 0.7 (1.6; 0–12).

The item frequencies are listed in Table 1. Only 32.5% and 31.1% disagreed with the statements that COVID-19 vaccination had strong side effects and low protectiveness prior to the fifth wave outbreak, respectively. Only 14.9% disagreed with the statement that they were at low risk of contracting COVID-19 prior to the fifth outbreak. It is also observed that 46.5% had frequently/always been exposed to news reporting post-vaccination deaths of older people (sometimes: 43.9%) prior to the fifth wave outbreak; 59.6%, 59.4%, and 52.3% found the information about older people’s suitability to take up vaccination given by the local health experts, mass/social media, and the local government conflicting/inconsistent, respectively. Of the participants, 38.3% agreed/strongly agreed that they had received counter information that older people were not suitable for vaccination from mass/social media (in addition to 44.9% who were uncertain, i.e., neither agreed nor disagreed with the statement); 2.6% agreed/strongly agreed that health professionals had told them that they were not suitable to take up COVID-19 vaccination (in addition to 34.8% who were uncertain, i.e., neither agreed nor disagreed with the statement). Regarding the family subjective norm, 8.2% found that their family was unsupportive towards their COVID-19 vaccination (in addition to 31.4% neither agree nor disagree). Regarding vaccination decision making of the participant’s vaccination, 75.8%, 20.7%, and 3.5% self-reported that the decision to take up COVID-19 vaccination was made mainly by the participants themselves, mutually by the participant and his/her family members, and mainly by the family members, respectively. The level of probable mild-to-moderate depression (PHQ-9 ≥ 5) was 3.8%.

### 3.2. Background Factors Associated with the First Dose Vaccination at Phase 3 Versus Phase 1 and Versus Phase 2

The findings are presented in Table 2. People vaccinated at Phase 3 were more likely than those vaccinated at Phase 1 to be aged 71–80 years (versus aged 60–70; ORc = 3.23, 95% CI: 1.62, 6.44) and >80 years (versus aged 60–70; ORc = 2.45, 95% CI: 1.05, 5.73), having an educational level of secondary school (versus college or above; ORc = 5.34, 95% CI: 1.16, 24.60) and primary school or below (versus college or above; ORc = 8.75, 95% CI: 1.85, 41.90), not currently married (versus currently married; ORc = 2.46, 95% CI: 1.22, 4.96), being retired (versus full time employment; ORc = 8.59, 95% CI: 1.88, 39.31), being a homemaker (versus full time employment; ORc = 11.88, 95% CI: 2.52, 56.00), and having chronic disease(s) (ORc = 2.12, 95% CI: 1.14, 3.95). People vaccinated at Phase 3 were more likely than those vaccinated at Phase 2 to be retired (versus full time employment; ORc = 4.96; 95% CI: 1.11, 21.12) andbe a homemaker (versus full time employment: ORc = 4.81; 95% CI: 1.07, 21.74). The other associations were statistically non-significant.

### 3.3. Associations between the Independent Variables and the Timing of the First Dose of COVID-19 Vaccination

The results of the multivariable logistic analysis (adjusted for the background factors) are shown in Table 3. Seven factors were significantly associated with both dependent variables (i.e., vaccination at Phase 3 versus Phase 1 and vaccination at Phase 3 versus Phase 2). They included: (a) a negative attitude towards COVID-19 vaccination prior to the fifth wave outbreak (ORa = 1.86 and 1.82, respectively), (b) a perceived low risk of COVID-19 infection prior to the fifth wave outbreak (ORa = 1.60 and 2.10, respectively), (c) exposure to conflicting information about the suitability of older people’s vaccination prior to the fifth outbreak (ORa = 1.39 and 1.35, respectively), (d) exposure to counter information that older people should not take up vaccination obtained from mass/social media prior to the fifth wave outbreak (ORa = 2.44 and 1.96, respectively), (e) counter information obtained from health professionals that he/she should not take up vaccination prior to the fifth wave outbreak (ORa = 2.00 and 4.53, respectively), (f) family’s unsupportive attitude towards COVID-19 vaccination prior to the fifth wave outbreak (ORa = 2.18 and 3.83, respectively), and (g) depressive symptoms (ORa = 1.35 and 1.29, respectively). Frequency of exposure to news reporting older people’s post-vaccination deaths prior to the fifth wave outbreak was positively associated with higher likelihoods in the Phase 3 versus Phase 2 comparison (ORa = 1.50), but not in the Phase 3 versus Phase 1 comparison. The two associations of the involvement of family members in the decision-making process were statistically non-significant (see Table 3). Similar univariate logistic regression results were obtained and presented in Table S1.

### 3.4. Comparing Perceptions Related to the Fifth Wave Outbreak between People Vaccinated at Phase 3 Versus Vaccinated at Phase 1 or 2

It is observed from Table 4 that people vaccinated at Phase 3 were more likely than those vaccinated at Phase 1 or 2 to have a perceived increase in worries about hospitalization or death related to COVID-19 (53.8% versus 48.7%; *p* < 0.001), perceived relationship between the numerous deaths of older people and low vaccination rate during the fifth wave outbreak (38.0% versus 29.0%; *p* = 0.025), and feel high/extremely high levels of emotional distress when the fifth outbreak occurred (42.5% versus 18.6%). The difference in worries about COVID-19 infection was close to statistical significance (61.3% versus 56.5%; *p* = 0.073).

## 4. Discussion

This study, which was conducted during the ongoing fifth wave outbreak of COVID-19 (June to July 2022) in Hong Kong, reported a prevalence of the first dose COVID-19 vaccination of 94.0% among community dwelling people aged 60 or older, as compared to the local data recording a prevalence of 84.6% in this age group as of 31 July 2022 [31]. Importantly, 21.3% of these vaccinated older people took their first dose during the severe fifth wave outbreak causing 6359 deaths among people aged 60 or older, which constituted about 70% of all COVID-19 related deaths in Hong Kong [31]. A previous local study conducted about one year prior to the fifth wave outbreak (May 2021) found that 19.7% of this age group refused to vaccinate (i.e., would not consider vaccinating at all) [12]; it is plausible that some of the previous refusers might have been motivated to take the first dose vaccination during the severe outbreak in Phase 3.

As multiple reasons exist, it is a limitation of the present study that we did not ask directly whether the outbreak caused them to take up vaccination. Nonetheless, the comparisons between the two groups vaccinated at Phase 3 versus Phase 1 or 2 may shed some insights. Those vaccinated at Phase 3 were more likely than those vaccinated at Phase 1/2 to have worries about COVID-19-related hospitalization/death, feel emotional distress when the outbreak occurred, and perceive a relationship between older patients’ deaths and low vaccination rate. Thus, it is inferred that the stronger increase in perceived severity and perceived susceptibility due to the severe fifth wave outbreak might have motivated some older people who were hesitant or refused to take COVID-19 vaccines to take their first dose vaccination at Phase 3. The contention is supported by the HBM [35].

Significant socio-demographic factors explaining the variations in the timing of the first dose vaccination were found. Phase 3 vaccinations were more likely than Phase 1 vaccinations to involve people aged >70 years, those having at least one chronic disease, primary or secondary school leavers, participants not working full-time (retired people and homemakers), and currently not married (e.g., widowhood). In general, older people and those with chronic diseases demonstrated higher vaccine hesitancy and worries about vaccine safety at the initial phase of the rollout [37], which might have prevented the participants to take up vaccinations at Phase 1. Primary/secondary school leavers might seek and update information about COVID-19 vaccination less frequently, as help-seeking was positively associated with education level [58,59], and hence demonstrated a higher vaccine hesitancy, deferring vaccination to Phase 3. People not working full-time (e.g., retired people) and in widowhood tended to be older and hence more likely to take up vaccination at Phase 3 instead of Phase 1 and 2. Future campaigns promoting vaccinations for new pandemics among older people should take these socio-demographic differences into account and focus on those of higher age and having less education. Employment status (retirement and homemakers) was the only significant socio-demographic factor that was significantly associated with vaccination at Phase 3 versus Phase 2, implying that the impact of the socio-demographic factors might mainly manifest at the initial phase of the rollout, and became less important six months afterwards.

It is observed that negative attitudes about vaccination prior to the fifth wave outbreak (strong side effects and low protectiveness) was quite common while perceived risk was quite low, possibly due to the low prevalence of COVID-19 infection in Hong Kong during that time period. High vaccine hesitancy was hence expected. Furthermore, such factors were significantly associated with Phase 3 vaccination versus Phase 1 and Phase 3 versus Phase 2 vaccination. Other contextual factors might have amplified the negative attitudes towards vaccination. For instance, mass and social media play important roles in influencing COVID-19 vaccination, especially at the early rollout phase [38]. In our case, 46.5% of the participants were frequently exposed to the news about post-vaccination deaths of older people soon after the vaccine rollout, and such exposure was significantly associated with vaccination at Phase 3 versus Phase 1 and versus Phase 2. Indeed, the local news about older people’s post-vaccination deaths, emphasizing their pre-existing chronic disease conditions, hit the news headlines soon after the vaccine rollout. The reports might have enhanced older people’s concerns about vaccine safety and hence increased their hesitancy and refusals. Furthermore, there was no subsequent clear official update about the accumulating evidence about the vaccines’ safety, although international recommendations that older people (including those with controlled chronic diseases) should take up COVID-19 vaccination was evident. In future pandemic involving novel vaccines, news about initial severe side effects of the vaccine should be handled prudently by the government and mass media to avoid negative attitudes and be consciously followed up with elaborations and clarification. 

One of the key reasons for not taking up vaccination was doubtful suitability about the vaccination [60]. Unclear and inconsistent information about the suitability of vaccination reduced motivation toward vaccination [37,44]. The information environment is important but was not favorable in Hong Kong, as the majority of the participants found the information about suitability provided by the local health experts and the government prior to the fifth wave outbreak unclear and conflicting, while such perceptions were significantly associated with vaccination at Phase 3 instead of Phase 1or Phase 2. First, some vocal and influential experts have expressed inconsistent views about vaccination safety and immediate vaccination among themselves and over time. Second, the government seemed to be lukewarm in promoting vaccine safety among older people and those with chronic disease prior to the fifth outbreak, although stronger messages were disseminated during the fifth outbreak. For instance, the official line given to the general public was often “consult your doctor if you have concerns about your suitability”. Yet, many older people in Hong Kong might not have access to a doctor’s advice regarding COVID-19 vaccination. A study conducted in Hong Kong found that recommendations from the government were the strongest cues motivating COVID-19 vaccination [60]. The government hence needs to take a stronger and clearer position about suitability and discuss with primary care physicians about how to deliver clear, consistent, and supportive messages, as well as work out a guideline at the beginning of vaccine rollout in future pandemics. 

The cue to action is a construct of the HBM which was associated with many health-related behaviors [35,61,62]. Advice given by health professionals was significantly and strongly associated with vaccination in general [63] and COVID-19 vaccination behavior [64]. Although only 2.6% of the participants agreed that they had been advised against their COVID-19 vaccination by health professionals prior to the fifth wave outbreak, close to 40% of them were indeed uncertain (neither agree nor disagree) about whether they had received such counter-information from health professionals, indicating the common presence of a negative cue to action (or absence of positive cue to action), and thus a less than ideal and inductive information environment. Counter information against vaccination among older people in mass/social media prior to the fifth outbreak was also common (over 50%), as shown by the participants’ responses. The overall scale about counter-information received was significantly associated with late vaccination at Phase 3 instead of Phase 1 or 2. Thus, to promote early vaccinations in future pandemics, health professionals and mass media need to reach a consensus and take a more active role in advising older people to take up the recommended vaccination. Social media campaigns delivering strong and evidence-based information about vaccine safety for older and chronically ill people at the early phase of new pandemics are greatly warranted. Establishing a governmental central hotline run by trained volunteers may be potentially effective.

Family influences are prominent for COVID-19 vaccination [37]. An unsupportive subjective norm in the family setting was observed among close to 10% of the participants but 31.4% seemed unclear about whether the family was unsupportive (neither agree nor disagree with the statement). As vaccine hesitancy was common prior to the fifth wave outbreak and at the early phase of the rollout [12,15,60], some family members might themselves be hesitant. Further research should look at the dynamics between family members’ subjective norm and the actual vaccination behavior. The findings highlight the importance of promoting older people’s vaccination to both the older people and their family members.

Depression in the general public during the pandemic period was common [51,52]. The prevalence in this sample was, however, quite low (3.8%). Previous studies have found a reduction in the prevalence of depression over time since the emergence of the pandemic [65,66]. The prevalence of depression in Hong Kong in April 2020 was 12.0% (based on the same tool) [67]; improvements might have occurred as the survey took place in July 2022, when the fifth outbreak has been mostly put under control and daily life became normal. However, depressive symptoms were significantly associated with late vaccination at Phase 3 instead of Phase 1 or 2. Health workers still need to promote mental health during the pandemic, and furthermore, focus on promoting early vaccination among older people showing depressive symptoms.

## 5. Limitations

First, this cross-sectional survey was unable to make causal or temporal inferences. Second, a recall bias may exist regarding items on situations prior to the fifth wave outbreak. Third, there may be a social desirability bias. The participants might tend to report favorable responses to perceptions/family influences on COVID-19 vaccination, which is a socially desirable behavior. Fourth, although the response rate was only 52.2%, it was comparable to other local population-based telephone surveys [15,68]. Fifth, some items were constructed for this study as related measurement tools are unavailable. Despite internal consistency, content validity, and the face validity of these scales/items tested in this study, future studies are needed for further validation. Last but not least, it is a limitation that this study did not ask about the occupational background, as some specific working experience (e.g., healthcare workers) may affect the timing of COVID-19 vaccination. In addition, other determinants of the timing of taking the first dose COVID-19 vaccination may exist but were not investigated in this study, including the impact of the vaccination pass requirement to enter public venues, perceived efficacy of local COVID-19 control measures, outcome expectancies of COVID-19 vaccinations, and trust towards health professionals/government.

## 6. Conclusions

This study observed a high prevalence of first-dose COVID-19 vaccination of 94.0% among people aged ≥60 in Hong Kong as of July 2022; the prevalence of a second-dose completion rate has also been high (87.0% as of July 2022) [39]. However, it is notable that the distribution of the first dose vaccination into three roughly six-month phases was about 27.7%, 51.1%, and 21.3%, respectively, which was in line with the normal distribution of various types of people adopting a novel behavior (innovator/early adopter: 16%; early majority/late majority: 68%; laggard: 16%) [69]. The Diffusion of Innovations theory has been applied to understand COVID-19 vaccination behavior [11]. It is expected that not everyone would take up a novel vaccination immediately. The important public health question is how to compress the time distribution, i.e., to motivate some of the late majority and laggards to take up the vaccination sooner, which would avoid numerous unnecessary deaths and hospitals. The speeding up of vaccination at the population level is especially important for countries where treatments are not available to all patients. 

The significant factors found in the present study have important public health implications for present and future pandemics. First, the clarification and consistency of information given by local health professionals and the government, as well as the timely rectification of the misinformation count. Second, the government and health professionals need to take more proactive roles and strong, unified, and consensual efforts to convince the laggards that vaccination among older and/or chronically ill people is safe according to the accumulative scientific evidence and international guidelines. Clear and consistent persuasive recommendations are needed. Third, detection and surveillance of any common misinformation emerging in the key mass/social media and immediate rectifications are warranted. Fourth, campaigns for early vaccination should target both older people and their family members. Last but not least, attention should be paid to vaccination among older people having depressive symptoms.

It is tragic that Hong Kong had over 9000 deaths due to COVID-19 within several weeks in the fifth-wave outbreak, out of whom 6359 (about 70%) were older unvaccinated individuals [31], despite the ownership of an advanced and high-quality medical system and rich experience in treating SARS patients. The findings have special implications for countries where older people’s vaccination rates were low, with treatments not readily available. The Hong Kong experience hence presents a painful but important lesson to the world. Other countries may have similar lessons to offer. A future modeling exercise about the impact on timing and distribution regarding vaccination and the number of COVID-19-related deaths would also be insightful. 

## Figures and Tables

**Table 1:** Participants’ characteristics (*n* = 376).

	*n*	%
**Background factors**		
Sex		
Female	245	65.2
Male	131	34.8
Age group (years)		
60–70	209	55.6
71–80	113	30.1
>80	54	14.4
Education level		
Primary school or below	143	38.0
Secondary school	198	52.7
College or above	32	8.5
Missing data	3	0.8
Current marital status		
Married	291	77.4
Others: single/separated/divorced/widowed/cohabitation	85	22.6
Employment status		
Full time	41	10.9
Part-time	26	6.9
Retired	175	46.5
Homemaker	130	34.6
Missing data	4	1.1
Living with other people (e.g., spouse, children, and siblings)		
Yes	330	87.8
No	46	12.2
Chronic disease status (as told by doctors)		
No/do not know	141	37.5
Yes	235	62.5
Previous COVID-19 infection		
No	279	74.2
Yes	97	25.8
**Timing of taking the first dose of COVID-19 vaccination**		
Phase 1	104	27.7
Phase 2	194	51.1
Phase 3	78	21.3
**Perceptions prior to the fifth wave outbreak**		
Perceived strong side effect of COVID-19 vaccination		
Strongly disagree	24	6.4
Disagree	98	26.1
Neutral	136	36.2
Agree	96	25.5
Strongly agree	22	5.9
Perceived low protectiveness of COVID-19 vaccination		
Strongly disagree	21	5.6
Disagree	96	25.5
Neutral	180	47.9
Agree	69	18.4
Strongly agree	10	2.7
Perceived low risk of COVID-19 infection		
Strongly disagree	15	4.0
Disagree	41	10.9
Neutral	90	23.9
Agree	188	50.0
Strongly agree	42	11.2
**Exposure to unsupportive information about suitability of older people’s vaccination prior to the fifth wave outbreak**		
Conflicting information about suitability of older people’s vaccination delivered by local health experts		
Strongly disagree	0	0.0
Disagree	60	16.0
Neutral	92	24.5
Agree	153	40.7
Strongly agree	71	18.9
Conflicting information about suitability of older people’s vaccination delivered by mass/social media		
Strongly disagree	0	0.0
Disagree	53	14.1
Neutral	100	26.6
Agree	154	41.0
Strongly agree	69	18.4
Conflicting information about suitability of older people’s vaccination delivered by the government		
Strongly disagree	0	0.0
Disagree	83	22.1
Neutral	96	25.5
Agree	125	33.2
Strongly agree	72	19.1
Counter information about old people’s suitability provided by media/social media		
Strongly disagree	8	2.1
Disagree	55	14.6
Neutral	169	44.9
Agree	93	24.7
Strongly agree	51	13.6
Counter information about the participant’s suitability provided by health professionals		
Strongly disagree	57	15.2
Disagree	178	47.3
Neutral	131	34.8
Agree	8	2.1
Strongly agree	2	0.5
Frequency of exposure to news reporting post-vaccination deaths of older people		
None at all	1	0.3
Rarely	35	9.3
Sometimes	165	43.9
Frequently	161	42.8
Always	14	3.7
**Family influences over COVID-19 vaccination prior to the fifth wave outbreak**		
Family’s attitude towards COVID-19 vaccination		
Strongly supportive	40	10.6
Supportive	187	49.7
Neutral	118	31.4
Unsupportive	26	6.9
Strongly unsupportive	5	1.3
Involvement of family members in the vaccination decision-making process		
Decision made mainly by the participant	285	75.8
Decision jointly made by the participant and family members	78	20.7
Decisions made mainly by family members	13	3.5
**Depressive symptoms**		
None	362	96.2
Mild	12	3.2
Moderate	2	0.6
Moderately severe	0	0.0
Severe	0	0.0

Note. Phase 3 refers to the six-month period during the fifth wave outbreak, i.e., February 2022 to July 2022. Phase 2 refers to the six-month period prior to the fifth wave outbreak, i.e., August 2021 to January 2022. Phase 1 refers to the first six months since vaccine roll-out, i.e., February 2021 to July 2021.

**Table 2:** Background factors of the timing of the first dose of COVID-19 vaccination.

	Participants Firstly Vaccinated at Phase 3 versus Phase 2 (*n* = 272)	Participants Firstly Vaccinated at Phase 3 versus Phase 1 (*n* = 184)
	ORc (95% CI)	ORc (95% CI)
Sex		
Female	Reference = 1.0	Reference = 1.0
Male	0.61 (0.34, 1.07)	0.75 (0.40, 1.41)
Age group (years)		
60–70	Reference = 1.0	Reference = 1.0
71–80	1.71 (0.96, 3.06)	3.23 (1.62, 6.44) **
>80	1.85 (0.88, 3.91)	2.45 (1.05, 5.73) *
Education level		
College or above	Reference = 1.0	Reference = 1.0
Secondary school	3.25 (0.71, 14.84)	5.34 (1.16, 24.60) *
Primary school or below	3.37 (0.73, 15.52)	8.75 (1.85, 41.39) **
Missing data	3.75 (0.22, 62.76)	NA
Current marital status		
Married	Reference = 1.0	Reference = 1.0
Others: single/separated/divorced/widowed/cohabitation	1.72 (0.96, 3.07)	2.46 (1.22, 4.96) *
Employment status		
Full time	Reference = 1.0	Reference = 1.0
Part time	3.00 (0.35, 25.46)	1.06 (0.13, 8.38)
Retired	4.96 (1.11, 22.12) *	8.59 (1.88, 39.31) **
Homemaker	4.81 (1.07, 21.74) *	11.88 (2.52, 56.00) **
Missing data	3.50 (0.24, 51.46)	NA
Living alone		
No	Reference = 1.0	Reference = 1.0
Yes	1.77 (0.83, 3.78)	1.25 (0.55, 2.83)
Chronic disease condition (as told by the doctors)		
No/do not know	Reference = 1.0	Reference = 1.0
Yes	1.42 (0.81, 2.51)	2.12 (1.14, 3.95) *
Previous COVID-19 infection		
No	Reference = 1.0	Reference = 1.0
Yes	0.96 (0.52, 1.77)	0.73 (0.38, 1.43)

Note. Phase 3 refers to the six-month period during the fifth wave outbreak, i.e., February 2022 to July 2022. Phase 2 refers to the six-month period prior to the fifth wave outbreak, i.e., August 2021 to January 2022. Phase 1 refers to the first six months since vaccine roll-out, i.e., February 2021 to July 2021. ORc = Crude odds ratio; CI = Confidence interval; NA = Not applicable. *, *p* < 0.05; **, *p* < 0.01.

**Table 3:** Multivariable logistic regression analysis adjusted for background factors.

	Participants Firstly Vaccinated at Phase 3 versus Phase 2 (*n* = 272)	Participants Firstly Vaccinated at Phase 3 versus Phase 1 (*n* = 184)
	ORa (95% CI)	ORa (95% CI)
**Perceptions prior to the fifth wave outbreak**		
Negative attitude towards COVID-19 vaccination	1.90 (1.52, 2.39) ***	1.81(1.41,2.32) ***
Perceived low risk of COVID-19 infection	1.85 (1.27, 2.68) **	2.44(1.60, 3.74) ***
**Exposure to unsupportive information about suitability of older people’s vaccination prior to the fifth wave outbreak**		
Conflicting information about suitability of older people’s vaccination	1.48 (1.29, 1.69) ***	1.54(1.31, 1.82) ***
Counter information about older people/participant’s suitability of vaccination provided by		
social media/mass media	3.03 (2.06, 4.46) ***	2.47(1.63, 3.75) ***
health professionals	2.02 (1.31, 3.11) **	3.87(2.17, 6.89) ***
Frequency of exposure to news reporting post-vaccination deaths of older people	1.64 (1.10, 2.46) *	1.13 (0.72, 1.76)
**Family influences over COVID-19 vaccination prior to the fifth wave outbreak**		
Family’s unsupportive attitude towards COVID-19 vaccination	2.16 (1.49, 3.15) ***	3.97 (2.31, 6.80) ***
Involvement of family members in the vaccination decision-making process		
Decision made mainly by participant	Reference = 1.0	Reference = 1.0
Decision jointly made by participant and family members	1.03 (0.50, 2.12)	0.59 (0.26, 1.34)
Decision made mainly by family members	0.87 (0.22, 3.50)	5.84 (0.34, 100.24)
**Depressive symptoms**	1.35 (1.13, 1.61) **	1.28 (1.03, 1.59) *

Note. Phase 3 refers to the six-month period during the fifth wave outbreak, i.e., February 2022 to July 2022. Phase 2 refers to the six-month period prior to the fifth wave outbreak, i.e., August 2021 to January 2022. Phase 1 refers to the first six months since vaccine roll-out, i.e., February 2021 to July 2021. ORa = Adjusted odds ratio; CI = Confidence interval. *, *p* < 0.05; **, *p* < 0.01. ***, *p* < 0.001. The models were adjusted for age group, sex, education level, current marital status, employment status, whether living with others (e.g., spouses and family members), chronic disease condition, and previous COVID-19 infection.

**Table 4:** Comparisons of perceptions and emotional distress between those firstly vaccinated at Phase 3 (during outbreak) versus vaccinated at Phase 1 or 2 (pre-outbreak).

	Time Taking Up the First Dose of COVID-19 Vaccination				
	Phase 3		Phase 1 or 2		*p* of χ^2^
	*n*	%	*n*	%	
Increase in worries about infection					0.073
Strongly disagree	4	5.0	7	2.4	
Disagree	22	27.5	75	25.3	
Neutral	5	6.3	47	15.9	
Agree	42	52.5	155	52.4	
Strongly agree	7	8.8	12	4.1	
Increase in worries about hospitalization or death related to COVID-19					<0.001
Strongly disagree	5	6.3	11	3.7	
Disagree	23	28.7	95	32.1	
Neutral	9	11.3	46	15.5	
Agree	26	32.5	134	45.3	
Strongly agree	17	21.3	10	3.4	
Perceived strength of association between death of elderly people and lack of vaccination during the fifth wave outbreak					0.025
Extremely weak	0	0.0	7	2.4	
Weak	22	27.5	58	19.6	
Moderate	26	32.5	145	49.0	
Strong	30	37.5	75	25.3	
Extremely strong	2	2.5	11	3.7	
Emotional distress when the fifth wave outbreak occurred					<0.001
Extremely low	1	1.3	22	7.4	
Low	24	30.0	119	40.2	
Moderate	21	26.3	100	33.8	
High	28	35.0	44	14.9	
Extremely high	6	7.5	11	3.7	

Note. Phase 3 refers to the period during the fifth wave outbreak, i.e., February 2022 to July 2022. Phase 2 refers to the six months prior to the fifth wave outbreak, i.e., August 2021 to January 2022. Phase 1 refers to the first six months since vaccine roll-out, i.e., February 2021 to July 2021.

## Data Availability

Data are available upon reasonable request.

## Supplementary Materials

The following supporting information can be downloaded at: https://www.mdpi.com/article/10.3390/vaccines11061029/s1, Table S1: Univariate logistic regression analysis.
