# Supplementary material for: Levels and Determinants of COVID-19 Vaccination at a Later Phase among Chinese Older People Aged 60 Years or Older: A Population-Based Survey
Source: Vaccines (Basel). 2023 May 26;11(6):1029. doi: 10.3390/vaccines11061029 (PMC10304556; doi:10.3390/vaccines11061029)
Supplement: Supplementary file 1 [file vaccines-11-01029-s001.zip › vaccines-2409193-supplementary.pdf]

Table S1. Univariate logistic regression analysis

	**Participants firstly vaccinated at Phase 3 versus Phase 2** (n = 272)	**Participants firstly vaccinated at Phase 3 versus Phase I** (n = 184)
	ORc (95% CI)	ORc (95% CI)
**Perceptions prior to the fifth wave outbreak**		
Negative attitude towards COVID-19 vaccination	1.86 (1.50, 2.31)***	1.82 (1.45, 2.27)***
Perceived low risk of COVID-19 infection	1.60 (1.15, 2.22)**	2.10 (1.48, 2.97)***
**Exposure to unsupportive information about suitability of older people's vaccination prior to the fifth wave outbreak**		
Conflicting information about suitability of older people's vaccination	1.39 (1.24, 1.56)***	1.35 (1.20, 1.52)***
Counter information about older people/participant's suitability of vaccination provided by		
social media/mass media	2.44 (1.77, 3.36)***	1.96 (1.43, 2.69)***
health professionals	2.00 (1.35, 2.98)**	4.53 (2.68, 7.67)***
Frequency of exposure to news reporting post-vaccination deaths of older people	1.50 (1.03, 2.17)*	0.95 (0.66, 1.39)
**Family influences over COVID-19 vaccination prior to the fifth wave outbreak**		
Family's unsupportive attitude towards COVID-19 vaccination	2.18 (1.53, 3.11)***	3.83 (2.43, 6.05)***
Involvement of family members in the vaccination decision-making process		
Decision made mainly by participant	Reference = 1.0	Reference = 1.0
Decision jointly made by participant and family members	1.23 (0.64, 2.36)	0.85 (0.42, 1.72)
Decision made mainly by family members	1.26 (0.37, 4.35)	5.22 (0.57, 47.94)
**Depressive symptoms**	1.35 (1.14, 1.59)***	1.29 (1.07, 1.56)***

Note. Phase 3 refers to the six-month period during the fifth wave outbreak, i.e., February 2022 to July 2022. Phase 2 refers to the six-month period prior to the fifth wave outbreak, i.e., August 2021 to January 2022. Phase 1 refers to the first six months since vaccine roll-out, i.e., February 2021 to July 2021. ORc = Crude odds ratio; CI = Confidence interval. *, $p<0.05$; **, $p<0.01$; ***, $p<0.001$.
